# Differentiating Grade in Breast Invasive Ductal Carcinoma Using Texture Analysis of MRI

**DOI:** 10.1155/2020/6913418

**Published:** 2020-04-07

**Authors:** Gaoteng Yuan, Yihui Liu, Wei Huang, Bing Hu

**Affiliations:** ^1^School of Computer Science and Technology, Qilu University of Technology (Shandong Academy of Sciences), Jinan 250353, China; ^2^Department VI of Radiation Oncology, Shandong Cancer Hospital and Institute, Shandong First Medical University and Shandong Academy of Medical Sciences, Jinan 250117, China; ^3^School of Medicine and Life Sciences, University of Jinan, Jinan 250022, China

## Abstract

**Purpose:**

The objective of this study is to investigate the use of texture analysis (TA) of magnetic resonance image (MRI) enhanced scan and machine learning methods for distinguishing different grades in breast invasive ductal carcinoma (IDC). Preoperative prediction of the grade of IDC can provide reference for different clinical treatments, so it has important practice values in clinic.

**Methods:**

Firstly, a breast cancer segmentation model based on discrete wavelet transform (DWT) and *K*-means algorithm is proposed. Secondly, TA was performed and the Gabor wavelet analysis is used to extract the texture feature of an MRI tumor. Then, according to the distance relationship between the features, key features are sorted and feature subsets are selected. Finally, the feature subset is classified by using a support vector machine and adjusted parameters to achieve the best classification effect.

**Results:**

By selecting key features for classification prediction, the classification accuracy of the classification model can reach 81.33%. 3-, 4-, and 5-fold cross-validation of the prediction accuracy of the support vector machine model is 77.79%~81.94%.

**Conclusion:**

The pathological grading of IDC can be predicted and evaluated by texture analysis and feature extraction of breast tumors. This method can provide much valuable information for doctors' clinical diagnosis. With further development, the model demonstrates high potential for practical clinical use.

## 1. Introduction

Breast cancer (BC) is one of the most common malignances in women [1]. The recent survey found that the occurrence rate grows rapidly in China, especially in developed regions. The most common histological type of breast cancer is invasive or infiltrating ductal carcinoma (IDC), which accounts for up to 70% of all BC cases. At present, the most common current method for histological grading of IDC is the “Elston and Eills method,” which is the latest modification of the “Bloom and Richardson method” [2]. There are two kinds of IDC treatment: breast conserving surgery and total mastectomy. Different grades of IDC correspond to different treatments. IDC grade diagnosis is usually established using stereotactic biopsy. Preoperative prediction of the grade of invasive ductal carcinoma can provide reference for doctors' treatment [3]. Although magnetic resonance imaging (MRI) can describe an IDC tumor, it is impossible to predict the IDC grades.

Image-based IDC characteristics include lesion size, imaging signal intensity, degree and method of image enhancement, and paratumor edema. In recent years, the development of MRI technology is rapid. In particular, the application of fat suppression technology and contrast enhancement greatly improves the sensitivity and specificity of MRI in the diagnosis of breast tumors [4]. MRI can provide a good image of soft tissue and can clearly distinguish an IDC tumor and the invasion range of surrounding tissue. It is of great research value to predict the grade of IDC by analyzing the specific areas of MRI. The goal of this study is to provide an automated tool that may assist in the imaging evaluation of breast neoplasms by evaluating the IDC grade. These issues are of critical clinical importance in making decisions regarding initial and evolving treatment strategies, and conventional MRI is often not adequate in providing answers. Automated tools, if proven accurate, can ultimately be applied to provide more reliable differentiation. So, it has great clinical significance for diagnosis and treatment.

Texture analysis (TA) is an advanced image processing method for extracting and quantifying features related to local patterns in images [5]. TA is a quantitative and systematic approach over a large range of spatial frequencies, giving it the potential to outperform expert visual pattern analysis to MRI and yielding promising results for the grades of IDC. There are lots of similar research on MRI texture analysis and machine learning at the moment. For example, Zacharaki et al. [6] used a computer-assisted classification method combining MRI and machine learning, and they developed and used it for differential diagnosis of brain tumor. But this method needs the experience of doctors to provide reference. This method lacks practicability. In Ref. [7], Nayak et al. propose a new automatic computer-aided diagnosis (CAD) which is based on discrete wavelet transform (DWT) and random forests to classify brain MRI. The results of the experiments reveal that the proposed scheme is superior to other state-of-the-art techniques in terms of classification accuracy, with a substantially reduced number of features. It shows that the method of wavelet analysis can analyze a tumor image. However, this method still needs the assistance of doctors and is not practical. At the same time, there are also studies using deep learning to predict tumor types. Kooi et al. [8] applied a convolutional neural network to the recognition of malignant lesions of breast cancer. This method can achieve better recognition results at low sensitivity in comparison with traditional computer-aided methods, and the accuracy rate of this method will be higher at high sensitivity. But this method needs a large amount of data set training, so it is limited to a certain extent due to the difficulty in data collection. Medical data is characterized by a small amount of data and lack of prior knowledge. So it is not suitable for deep learning. In Ref. [9], Liu et al. established a support vector machine (SVM) classification model which is based on the Gabor wavelet TA to predict the primary central nervous system lymphoma (PCNSL) and glioblastoma multiforme (GBM). The result shows that the model can distinguish different diagnosis categories of tumor images. It shows that the Gabor analysis of MRI can distinguish different types of tumors. But this method is used to predict two kinds of tumors with different densities and textures. It cannot be proved that this method can predict different grades of tumor. Li et al. [10] used a variety of texture analysis methods combined with a machine learning classification model to explore the classification of lung cancer brain metastasis. This method shows that TA may predict the differences among various pathological types of lung cancer with brain metastases. These studies show that the Gabor features can distinguish different types of tumors. At present, there are few researches on IDC grade prediction. It is of great value to build an IDC grade prediction model by analyzing breast MRI.

In this paper, data samples were constructed by collecting MRI and pathological results of IDC patients before operation. We selected the focus area of the MRI of tumors. DWT and the Gabor wavelet are used to analyze the tumor area and obtain the texture features of the image [11]. The linear discriminant analysis (LDA) method is used to analyze the features and obtain several key features. Then, the support vector machine (SVM) model is used to classify features and build a prediction model. Experimental results show that our model can evaluate different grades of invasive ductal carcinoma.

## 2. Data and Methods

**Figure 1:**
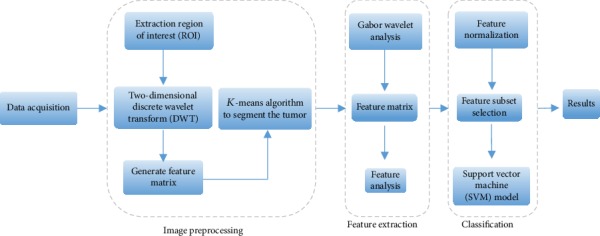
Block diagram of the proposed scheme.

We propose a model based on TA and machine learning methods for IDC grade prediction. In this paper, we use the Gabor wavelets to extract texture features from MRI. The Gabor wavelet with different directions and frequencies can detect slight differences between grades of IDC. Firstly, a breast cancer segmentation model based on DWT and the *K*-means algorithm is proposed. Secondly, TA was performed, and the Gabor wavelet analysis was used to extract the texture feature of MR images [12]. Then, according to the distance relationship between the features, key features are sorted and feature subsets are selected. Finally, the support vector machine model is used to classify feature subsets, and the prediction model is constructed.  Figure 1 demonstrates the overall block diagram of the proposed scheme.

### 2.1. Data Acquisition

We collected 28 IDC patients from Shandong Cancer Hospital as research data. All patients underwent biopsy or surgical resection of the tumor with histopathological diagnosis. The pathological results of these patients were based on the Elston and Eills methods. These patients were histologically diagnosed and graded based on the Elston and Eills method as 14 grade III IDC patients and 14 grade III IDC patients. (Because most of the patients with breast IDC are at or above grade II when they are diagnosed, the data of the grade I patients are less.) On average, 4~15 MRI sections were selected for each patient. All the patients were female, 29~63 years old, with an average age of 46 years. These patients had not been treated at the time of MRI.

**Figure 2:**
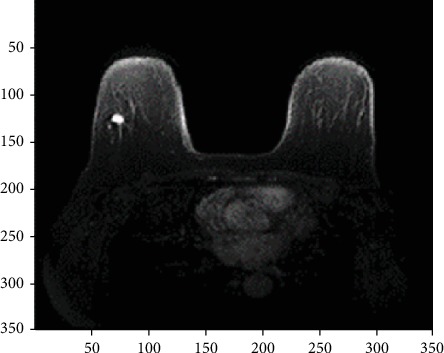
Enhanced sequence MR image of IDC.

The Philips Achieva 3.0T field strength MR scanner was used for breast examination. For each MR image, an enhanced sequence, 2.2 ms echo time (TE), 4.4 ms repetition time (TR), and 3 slices whose diameters are equal to or larger than 1.5 cm are selected for calculating the combined texture features to evaluate performance.  Figure 2 shows the enhanced sequence MR image of an infiltrating ductal carcinoma of the breast.

### 2.2. Image Preprocessing

**Figure 3:**
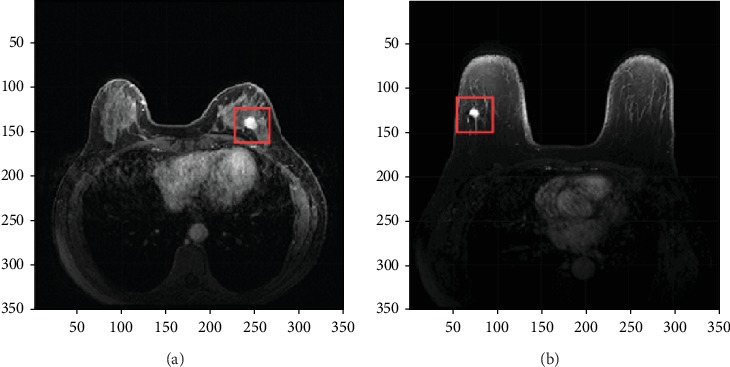
Enhanced sequence MR images: (a) slice of a grade II IDC and (b) slice of a grade III IDC. The red rectangular areas represent the lesion ROI.

The preprocessing of MRI is an important step in extracting texture information from tumor areas, involving denoising, extraction of the region of interest (ROI), segmentation of the effective determining area, etc. The original MRI matrix size is 352∗352, and we select a 60∗60 matrix around the lesion area as the region of interest (ROI). Figures 3(a) and 3(b) show slices of enhanced sequence MR images.  Figure 3(a) shows a slice of a grade II IDC, and Figure 3(b) shows a slice of a grade III IDC. The red rectangular areas represent the lesion ROI.

**Figure 4:**
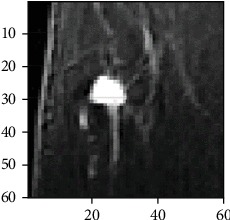
MR image of the ROI area of IDC.

In order to reduce mistaken recognition resulting from segmentation, the system adopted the two-dimensional discrete wavelet transform (DWT) to eliminate the noise of MRI [13]. DWT is a powerful tool for feature extraction as it allows analysis of images at various levels of resolution. The main advantage of wavelet is that it provides information on time-frequency localization of an image which is very important for segmentation [14].  Figure 4 shows the ROI area of IDC, which needs wavelet for decomposition.

**Figure 5:**
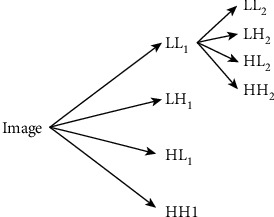
Decomposition process of DWT.

The basic idea of DWT is to decompose the original signal into a series of subband signals with different spatial resolutions and different frequency characteristics by stretching and translation. In the case of MR images, DWT is applied to each dimension individually. As a consequence, four subband images are obtained at each level. The four subband images are LL (low-low), LH (low-high), HL (high-low), and HH (high-high). From these, three subband images, namely, LH, HL, and HH, are the detailed (high-frequency) components in the horizontal, vertical, and diagonal directions, respectively. The LL subband images are the approximation (low-pass) component which is used for the next level DWT calculation [9]. The DWT decomposition process is shown in Figure 5.

**Figure 6:**
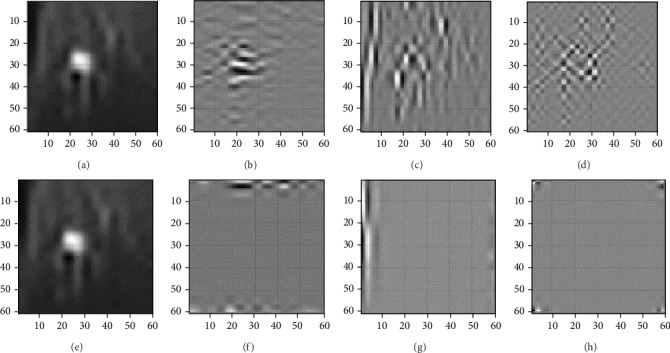
Wavelet approximation and details: (a) approximation at the 1st level, (b) horizontal detail at the 1st level, (c) vertical detail at the 1st level, (d) diagonal detail at the 1st level, (e) approximation at the 2nd level, (f) horizontal detail at the 2nd level, (g) vertical detail at the 2nd level, and (h) diagonal detail at the 2nd level.

After DWT decomposition at the 2nd level is performed on the ROI, the approximation at the 2nd level is obtained to combine the original image for the segment of the tumor.  Figure 6 shows the wavelet approximation and details in the horizontal, vertical, and diagonal directions at the 1st and 2nd levels of wavelet decomposition [15].

The tumor area must be segmented for its TA to be accurately calculated. The difference of pixel value between the tumor area and the normal tissue on an MR image is very obvious. However, MR images did not mark tumor areas and normal areas. Therefore, the method of supervised learning cannot be used to segment the tumor area. In order to solve this problem, we use the *K*-means algorithm to segment the tumor region. The *K*-means algorithm does not need prior knowledge to segment the tumor area. At the same time, the algorithm can combine the features of the MR image after wavelet decomposition.

*K*-means is a clustering algorithm based on distance similarity. By comparing the similarity between samples, the samples of the same form are divided into the same category [14]. The commonly used distance calculation methods are the Euclidean distance and the Manhattan distance. Because the MR image has been preprocessed, there is no abnormal value in image pixel. Considering the segmentation efficiency, we use the Euclidean distance as the difference measure.

There is a set of *n* vectors *X*_*j*_, and *j* = 1, ⋯, *n* is divided into *c* groups *G*_*i*_, *i* = 1, ⋯, *c*. The cost function is calculated based on the Euclidean distance between a vector *X*_*k*_ in group *j* and the corresponding cluster center *C*_*i*_ as follows:

(1) $$\begin{array}{c} J=\overset{c}{\underset{i=1}{\sum }}J_{i}=\overset{c}{\underset{i=1}{\sum }}\left(\underset{k,X_{k\in G_{i}}}{\sum }{\left\|X_{k}-C_{i}\right\|}^{2}\right). \end{array}$$

Here, *J*_*i*_ represents the cost function in grouping *i*.

The distinguished grouping can be defined as a binary membership matrix *U* of *c*∗*n*. The element *u*_*ij*_ is assigned a value of 1 or 0. When the *j*th data point *X*_*j*_ belongs to grouping *i*, *u*_*ij*_ is 1. Otherwise, it is 0. Once the cluster center *c*_*i*_ is identified, the minimum *U*_*ij*_ of formula (1) is pushed out:

(2) $$\begin{array}{c} u_{ij}=\left\{\begin{array}{cc} 1, & \text{if }{\left\|X_{j}-C_{i}\right\|}^{2}\leq \left\|X_{j}-C_{k}\right\|,\;k\neq i,\\ 0, & \text{otherwise}. \end{array}\right. \end{array}$$

Equation (2) can be interpreted as follows: if *C*_*i*_ is the center point closest to *X*_*j*_ among all cluster centers, then *X*_*j*_ belongs to group *i*.

On the other hand, if the membership function, for example, *u*_*ij*_, is determined, then the optimal center *C*_*i*_, i.e., the minimum of equation (1), is the average of all vectors in group *i*:

(3) $$\begin{array}{c} C_{i}=\frac{1}{\left|G_{i}\right|}\underset{k,X_{k}\in G_{i}}{\sum }X_{k}. \end{array}$$

Here, |*G*_*i*_| is the size of *G*_*i*_, or |*G*_*i*_| = ∑_*j*=1_^*n*^*u*_*ij*_.

The algorithm is presented by the pixels *X*_*i*_, *i* = 1, ⋯, *n*. It depends on the iteration of clustering center *C*_*i*_ and membership matrix *U*. The specific steps are as follows:

**Step 1 .**

Initialize the cluster center *C*_*i*_, *i* = 1, ⋯*C*. This is usually a random selection of four data points from all data points.

**Step 2 .**

Determine the membership matrix *U* by formula (2).

**Step 3 .**

Calculate the cost function according to formula (1). Stop if it is below a certain tolerance or if it is below a certain threshold compared with the previous iteration.

**Step 4 .**

Upgrade the cluster center according to formula (3), then go to Step 2.

**Figure 7:**
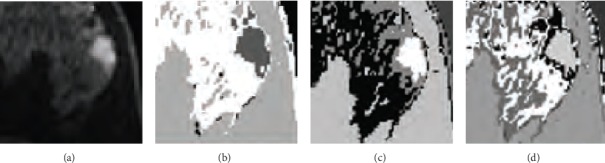
The *K*-means clustering results: (a) original image, (b) clustered into 4 categories, (c) clustered into 5 categories, and (d) clustered into 6 categories.

The performance of the *K*-means algorithm depends on the initial position of the cluster center, so it is necessary to run the algorithm several times because there will be a different set of initial cluster centers each time. We chose several clustering centers for the experiment. The optimal segmentation results are obtained by comparison. We choose 4, 5, and 6 cluster centers. The experimental results are shown in Figure 7. Because of the large difference between the tumor area and surrounding tissue pixels, each clustering can segment a tumor area.

**Figure 8:**
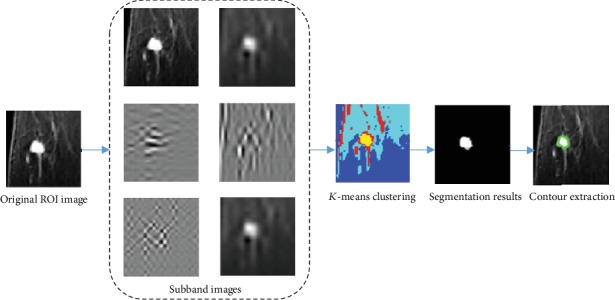
Segmentation process of tumor. The subbands of the horizontal, vertical, and diagonal directions of the wavelet decomposition; the approximate components of the 2nd level decomposition; and the original image are selected for *K*-means clustering.

The different subbands after wavelet decomposition are clustered to get the segmented tumor image [16].  Figure 8 shows the segmentation process. The outline of the tumor can be obtained by using this model.

### 2.3. Feature Extraction

This section presents the Gabor wavelet analysis of the ROIs of a tumor image for extracting the texture features [12]. The Gabor wavelets have a tunable orientation, radial scale bandwidths, and tunable center scales, allowing them to optimally achieve joint resolution in the spatial and frequency domains. Due to the Gabor wavelets capturing the local structure corresponding to spatial frequency (scales), spatial localization, and orientation selectivity, they are widely applied in many research areas, such as texture analysis and image segmentation [9, 17].

The impulse response of the Gabor filter can be defined as a cosine wave multiplied by a Gauss function. Because of the multiplicative convolution property, the Fourier transform of a Gabor filter impulse response is the convolution of its harmonic function Fourier transform and the Gabor function Fourier transform. The filter consists of a real part and an imaginary part, which are orthogonal to each other. The filter can be defined as follows:

(4) $$\begin{array}{c} \left\{\begin{array}{c} g{\left(x,y,\lambda ,\theta ,\varphi ,\sigma ,\gamma \right)}_{\text{real}}=e^{-\left(\left({x^{\prime }}^{2}+\gamma^{2}{y^{\prime }}^{2}\right)/2\sigma^{2}\right)}\cos\left(2\pi \frac{x^{\prime }}{\lambda }+\varphi \right),\\ g{\left(x,y,\lambda ,\theta ,\varphi ,\sigma ,\gamma \right)}_{\text{imag}}=e^{-\left(\left({x^{\prime }}^{2}+\gamma^{2}{y^{\prime }}^{2}\right)/2\sigma^{2}\right)}\sin\left(2\pi \frac{x^{\prime }}{\lambda }+\varphi \right), \end{array}\right. \end{array}$$

where

(5) $$\begin{array}{c} \left\{\begin{array}{c} x^{\prime }=x\cos\theta +y\sin\theta ,\\ y^{\prime }=-x\sin\theta +y\cos\theta , \end{array}\right. \end{array}$$

and *λ* is the wavelength, which can affect the filter scale (*λ* ≥ 2). *θ* is the direction of the filter and *φ* is the phase shift (‐180° ≤ *φ* ≤ 180°). *γ* is the spatial aspect ratio, and the shape of the filter is determined (*γ* = 1, the filter is circular); *σ* is the bandwidth that determines the variance of the Gauss filter (*σ* = 2*π*).

Image texture features can be extracted by convolving the image *M*(*x*, *y*) with the Gabor filters:

(6) $$\begin{array}{c} g\left(x,y,f,\theta \right)=M\times \varphi \left(x,y,f,\theta \right). \end{array}$$

**Figure 9:**
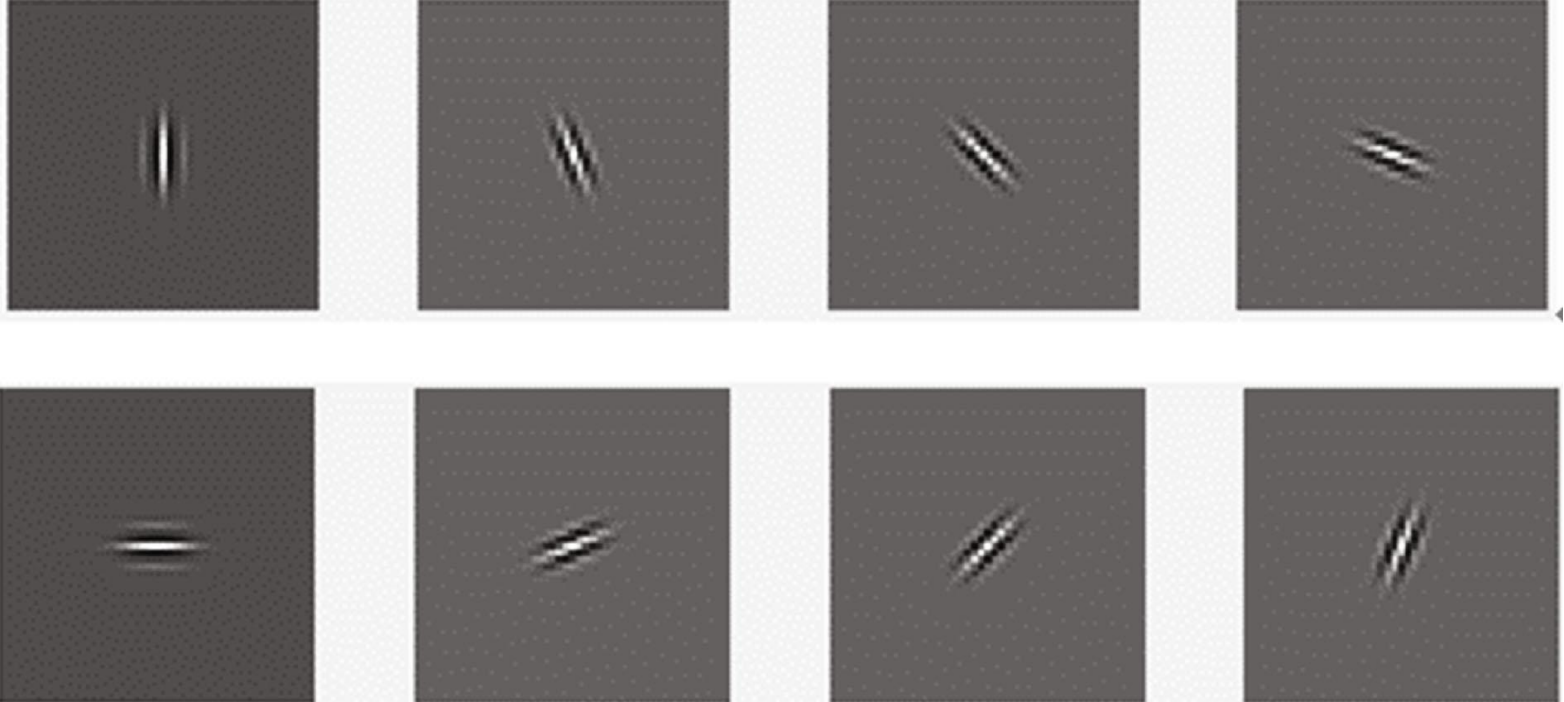
The two-dimensional Gabor wavelet function in different directions.

The Gabor filters with different frequencies *f*_*i*_ and orientations *θ*_*j*_ are selected to obtain the texture features of the tumor area.  Figure 9 shows a set of the Gabor wavelet functions with uniform scales (*λ* = 2) and different directions, with directions of 0°, 22.5°, 45°, 67.5°, 90°, 112.5°, 135°, and 157.5°, respectively.

**Figure 10:**
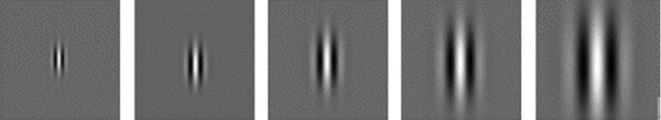
The two-dimensional Gabor wavelet function at different scales.

Figure 10 shows a set of the Gabor wavelet functions with the same direction (*φ* = 0°) and different scales, with wavelengths of 2, $2\sqrt{2}$, $2\sqrt{3}$, 4, and $2\sqrt{5}$, respectively.

**Figure 11:**
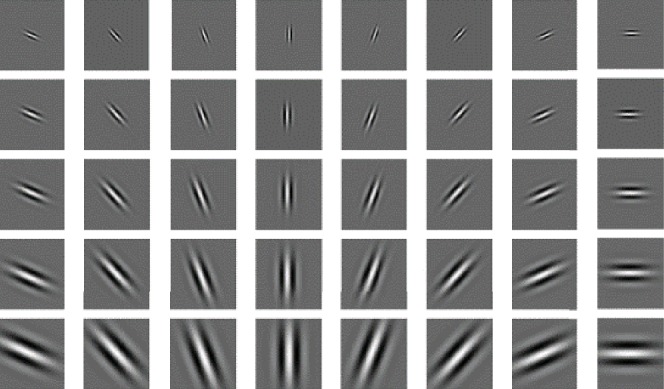
The five scale, eight-direction Gabor wavelet function.

In the process of generating the Gabor filter banks, the selection of direction and scale is a crucial step. As shown in Figure 11, the Gabor wavelet functions with five scales and eight directions are selected.

**Figure 12:**
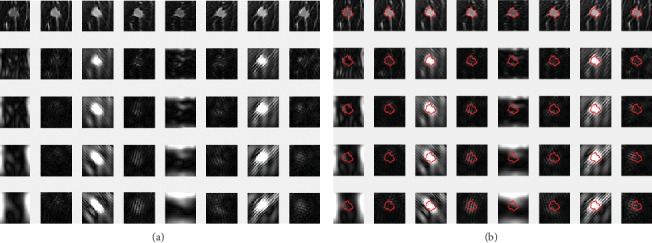
The Gabor-filtered image: (a) the Gabor wavelet-filtered image with five scales and eight directions and (b) tumor location markers in characteristic images.

The above 40 Gabor filter banks are used to filter the ROI of breast cancer MR images, and the filtering effect is shown in Figure 12(a).

In the stage of image pretreatment, we obtained the coordinates of the tumors on the MR images. According to the coordinates of the ROI, 40 feature maps after the Gabor transformation are marked in turn. Part of the feature image coordinate markers are shown in Figure 12(b).

The filtered image shows that the difference between the tumor area and the normal tissue is obvious. Therefore, we choose the mean value of the tumor area as the feature. Three MR images were selected for each patient, and 40 features could be obtained from one MR slice. So 123 features could be obtained from each patient combined with the original MR images. The features of each patient are calculated as follows:

(7) $$\begin{array}{c} F_{j}=\frac{1}{n}\sum I_{\left(x,y\right)}, \end{array}$$

where *F*_*j*_ is the feature value of each patient, *j* represents the number of 123 feature images for each patient (*j* = 1, 2, ⋯, 123), and *I*(*x*, *y*) is the pixel value of the image at the (*x*, *y*) coordinate. *n* represents the number of pixels in the tumor focus area of the patient.

**Figure 13:**
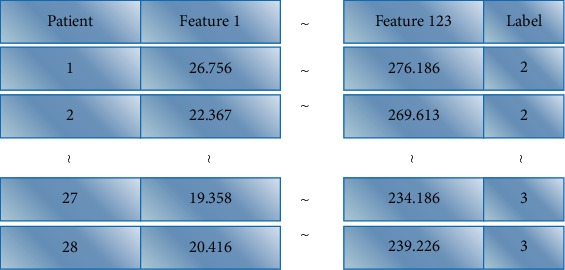
Feature matrix diagram.

According to the above steps, the corresponding features of patients are extracted. These features are constructed into feature matrices and tagged with pathological results. The feature matrix is shown in Figure 13.

### 2.4. Feature Analysis

**Figure 14:**
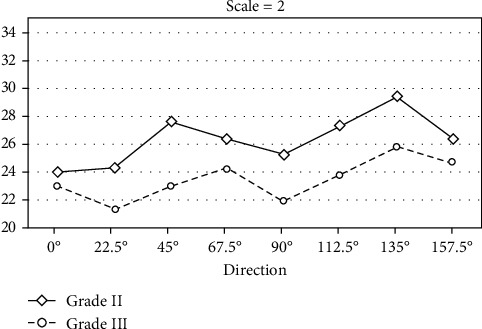
When *λ* = 2, it corresponds to the mean of all features in eight directions.

After obtaining the features of all samples, we need to further analyze the extracted features. We have calculated the mean values of all features at two grades in different directions and scales in turn. We hope to find out the difference between two grades of IDC through such a method.  Figure 14 below describes the mean of all features in eight directions when the scale is 2.

**Figure 15:**
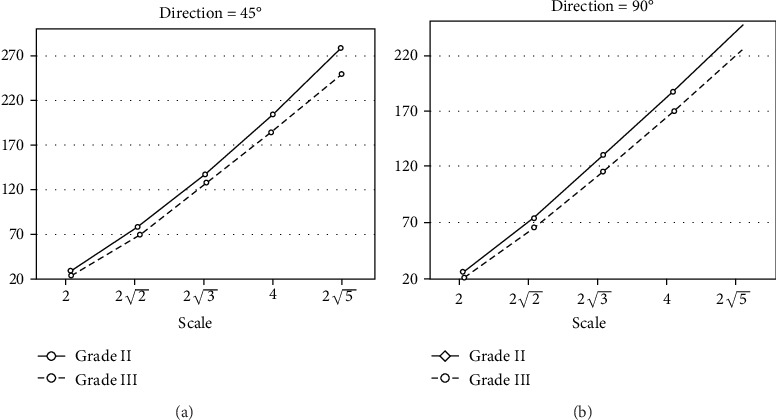
The mean values of five scale features correspond to two directions: (a) means of five scale characteristics at 45°direction and (b) means of five scale characteristics at 90°direction.

It can be seen from Figure 14 that in different directions, the average value of grade II is greater than grade III. In addition to direction comparison, we also compare two grades of IDC at different scales.  Figure 15 shows the mean values of all features at five scales corresponding to 45° and 90° degrees of orientation.

It can be seen that the mean of grade III IDC features is generally lower than that of grade II IDC features. It shows that there are differences in the Gabor texture between the two grades of tumors. Therefore, the IDC grade can be distinguished by texture analysis of the tumor ROIs in MR images.

We carry out the Gabor wavelet filtering with 5 scales and 8 directions. In some dimensions and directions, some features are not effective. There are even some features, because the feature value of individual patients is particularly large, which will produce wrong results. In order to avoid the situation of too long training time and data redundancy in the construction of a classification model, we need to reduce the number of features, improve the accuracy of the model, and simplify the model. We need to filter the features and select some of the most effective features. We use feature subset selection for 123 features to optimize the classification model.

We use the linear discriminant analysis (LDA) algorithm to sort the features. LDA is a method to get the optimal feature subset by sorting the minimum distance between the inner class and the maximum distance between the outer class. Generally, patterns of different classes can be distinguished because the domain of the classes in the feature space is different. Therefore, the smaller the overlap or no overlap, the better the separability of the categories [18]. We use distance to construct the separability criterion of categories. The distance from the point to the point set is used to select the feature. The formula is as follows:

(8) $$\begin{array}{c} D\left(x,\left\{a^{i}\right\}\right)=\sqrt{\frac{1}{K}\overset{K}{\underset{i=1}{\sum }}\left[\overset{n}{\underset{k=1}{\sum }}{\left(x_{k}-a_{k}^{i}\right)}^{2}\right]}. \end{array}$$

Assuming that there are *K* points in point set {*a*^*i*^}, *a*_*k*_^*i*^ denotes the *k* component of point *i* in the point set. The distance between the selected feature and the previously selected feature is expressed by dist; set the weight factor of {*a*^*i*^} feature to be expressed by *β*. The following formulas are used to calculate and rank the obtained values to obtain the optimal subset of several features.

(9) $$\begin{array}{c} \text{idx}=\sqrt{{\left(1-e^{-\left(\text{dist}/\beta \right)}\right)}^{2}}. \end{array}$$

**Figure 16:**
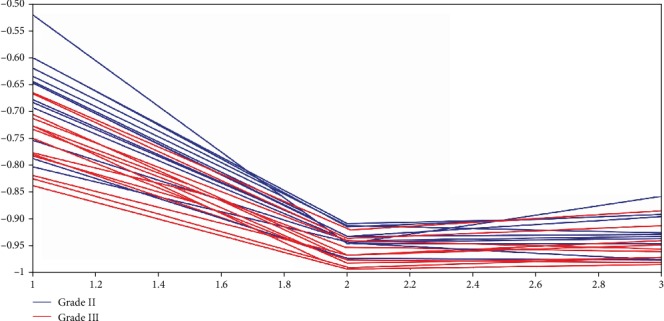
The key features selected based on $\varphi =45^{{}^{\circ}},\;\lambda =2\sqrt{2}$.

Sort the idx of 123 features, and the bigger the value, the more obvious the distinction is. We rank the features and select several key features.  Figure 16 is a line chart of two types of patients with the most obvious features ($\varphi =45^{{}^{\circ}},\;\lambda =2\sqrt{2}$). In this feature, most patients can be distinguished.

### 2.5. Support Vector Machine Classification Model

The support vector machine (SVM) is an important algorithm in machine learning and is widely used in the pattern recognition domain [19]. The main idea of SVM is to establish a hyperplane as a decision surface, which maximizes the isolation edge between positive and negative examples. The theory is mapping the linearly inseparable data in a low-dimension space to a high-dimension space and making it linearly separable. SVM has many unique advantages in solving small sample, nonlinear, and high latitude pattern recognition problems and can be applied to many machine learning problems. The SVM model includes four parts: feature selection, kernel function solution, threshold calculation, and decision function construction.

Select to divide the data into a training set and a test set, and set the training set as follows:

(10) $$\begin{array}{c} T=\left\{\left(x_{1},y_{1}\right),\cdots ,\left(x_{i},y_{i}\right)\right\}\in {\left(\text{X}\times Y\right)}^{l}, \end{array}$$

where *x*_*i*_ ∈ *X* = *R*^*n*^, *y*_*i*_ ∈ *Y* = {1, −1}, (*i* = 1, 2, ⋯, *l*), and *x*_*i*_ is the feature vector. Select proper kernel function *k*(*x*, *x*′) and proper parameter *C* to construct and solve the optimization problem:

(11) $$\begin{array}{c} \underset{\alpha }{\min}\frac{1}{2}\overset{j}{\underset{i=1}{\sum }}\overset{l}{\underset{j=1}{\sum }}y_{i}y_{j}\alpha_{i}\alpha_{j}K\left(x_{i},x_{j}\right)-\overset{l}{\underset{j=1}{\sum }}\alpha_{j}, \end{array}$$

where ∑_*i*=1_^*l*^*y*_*i*_*α*_*i*_ = 0, 0 ≤ *α*_*i*_ ≤ *C*, *i* = 1, ⋯, *l*, and the optimal solution can be obtained by the following formula:

(12) $$\begin{array}{c} \alpha^{*}={\left(\alpha_{1}^{*},\cdots ,\alpha_{l}^{*}\right)}^{T}. \end{array}$$

Select a positive component *α*^∗^ of 0 < *α*_*j*_^∗^ < *C* and calculate the threshold according to the component. The threshold calculation formula is as follows:

(13) $$\begin{array}{c} b^{*}=y_{j}-\overset{l}{\underset{i=1}{\sum }}y_{i}\alpha_{i}^{*}K\left(x_{i}-x_{j}\right). \end{array}$$

In addition, we need to construct a decision function to complete the final output. The decision function formula is as follows:

(14) $$\begin{array}{c} f\left(x\right)=\mathrm{sgn}\left(\overset{l}{\underset{i=1}{\sum }}\alpha_{i}^{*}y_{i}K\left(x,x_{i}\right)+b^{*}\right). \end{array}$$

The selection of the SVM kernel function is very important for its performance, especially for the linear and indivisible data. We refer to several key features obtained from feature analysis and classify them according to these features. With SVM, there is no uniform mode to choose SVM's kernel function and its parameters. Through constant debugging of parameters, the best classification effect is obtained. We choose different kernel functions and a different penalty factor *C* to classify. By adjusting the parameters and penalty factors of the kernel function, the best classification accuracy can be obtained.

**Figure 17:**
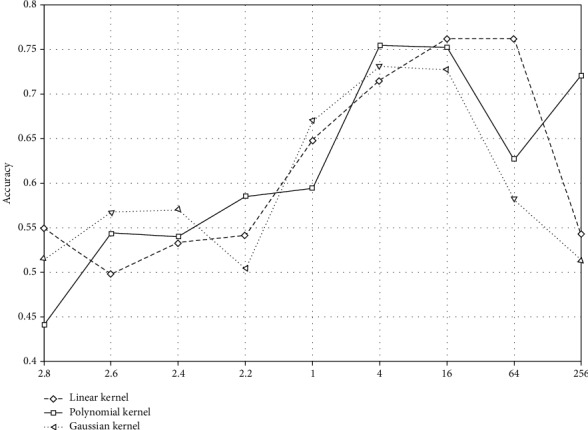
Penalty factor and precision line graph.

In view of the problems of SVM model parameter selection, the influence of penalty parameter and kernel function to SVM is analyzed. Six features are used for each classification.  Figure 17 is a line graph of the penalty factor *C* and the corresponding precision.

It can be seen from the figure that the penalty factor is of high precision from 1 to 64. Therefore, through further parameter optimization, we choose parameter *C* as 1, 2, 8, 16, and 32. The experimental results are shown in Table 1 (each parameter adjustment is verified by 3-fold cross-validations).

For the SVM method, through the different kernel functions, parameter analysis is used to establish the optimal kernel function and related parameters. The results show that the prediction accuracy can reach 81.33% by using the Gauss kernel function.

**Figure 18:**
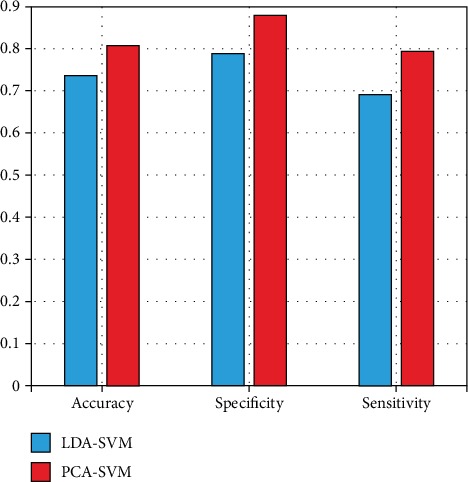
Comparison of PCA and LDA experimental results.

The above model is based on the feature extraction of the LDA algorithm. In order to further improve the accuracy of the model, we use the principal component analysis (PCA) algorithm to reduce the dimension of features. We hope to get the best model by comparing the two algorithms. The purpose of PCA is to use the idea of dimension reduction to transform multiple indexes into a few comprehensive indexes. This algorithm is suitable for large-scale data classification. However, the feature dimensions we extracted are only 123 dimensions, so the PCA algorithm is not as effective as LDA feature filtering after dimension reduction. The experimental results are shown in Figure 18.

## 3. Results and Discussion

After adjusting the parameters of related models, we need to further explore the impact of the number of key features on the classification accuracy. At the same time, it is not only necessary to compare the classification accuracy of the model but it is also necessary to use sensitivity and specificity to evaluate the performance of the model. The hardware used in this experiment is an Intel Core i7-6700 CPU @3.40 GHz with 16 GB of memory, and the software is Matlab2017b.

### 3.1. Results

**Figure 19:**
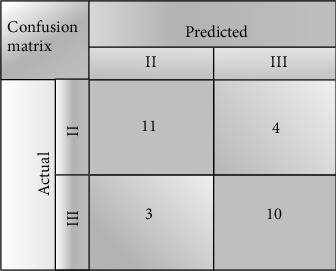
Classification results.

In the process of modeling, we have adjusted the parameters of SVM. We choose the best parameters to set our model. We use the confusion matrix to describe the classification results.  Figure 19 reflects the results of 3-fold cross-validation using 6 key features.

From the classification results, our model can distinguish these two grades of tumors. We also use sensitivity and specificity to evaluate the model. TP is used to represent the number of IDC grade III samples, and TN is used to represent the number of IDC grade 2 samples. *P* is used to represent the number of IDC grade III samples, and *N* is used to represent the number of IDC grade II samples. Let FP and FN be the number of false positive (IDC III) and false negative samples (IDC II), respectively. Accuracy is defined as accuracy = (TP + TN)/(*P* + *N*). Sensitivity is defined as sensitivity = TP/(TP + FN). Specificity is defined as specificity = TN/(TN + FP). We used 3, 4, 6, and 10 key features to classify them in turn. To further validate the experiment, we use 3-, 4-, and 5-fold cross-validation to get the best results. The results of classification prediction are shown in Table 2.

**Figure 20:**
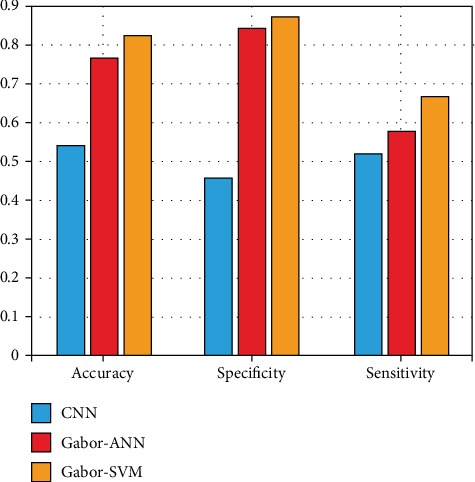
Comparison of various methods.

The above experiments show that the best classification result is obtained by selecting 3 or 8 key features. When the key feature was used for 3-, 4-, and 5-fold cross-validation experiments, 77.78%, 76.39%, and 76% accuracies were achieved, respectively. Accuracies of 80.55%, 81.94%, and 78.47% were achieved when 8 key features were selected. At the same time, we use the classification method of the convolutional neural network (CNN) to classify the grade of IDC [20]. Because of the small amount of data collected, the result of CNN classification is very poor. In addition, we use the artificial neural network (ANN) classifier to classify the Gabor extracted features. The experimental results are shown in Figure 20.

Experiments show that the classification effect of the Gabor wavelet combined with SVM is better than that of CNN and other classification methods. Our method is feasible.

Due to the lack of data, our initial model did not include IDC grade I. In order to verify the output of grade I IDC in the model, we selected two patients for the experiment. Multiple experiments showed that the output of the two patients was grade II. It is proven that our model can distinguish two grades of IDC of breast.

### 3.2. Discussion

It is well known that breast cancer has become an important disease endangering women's health. Patients in different situations have different treatment options. Therefore, the preoperative evaluation and prediction of breast cancer has great clinical significance. Invasive ductal carcinoma is the most common type of breast cancer. We analyzed the pathological results of breast cancer patients admitted to Shandong Cancer Hospital in recent three years. Among them, more than 2000 were ductal cancer patients, while only a few hundred were patients of other types of breast cancer, such as lobular carcinoma of the breast. So, the grade of IDC prediction can help most breast cancer patients. These materials indicate that preoperative prediction grade of invasive ductal carcinoma is of great clinical significance.

The characteristics of medical data are a small amount of data and no prior knowledge. At present, most of the disease classification models are processed text data. MR image data are rarely used for classification. It is very difficult to classify MR images by a single method. In order to solve this problem, we propose a combined model, which mainly involves the use of the Gabor wavelet to analyze MR images, extract features of different grades of IDC, and use the SVM model to complete feature classification. It is advantageous to build a classification model by using the combination of many methods. The results show that our scheme is feasible. Our model can provide reference for doctors' treatment plan. However, at this stage, the model still has some shortcomings, which need to be solved in the next work:

1. Because the grade of IDC is scored and evaluated by pathologists according to various indicators of pathological results, the results are not rigorous. In some cases, the results given by different doctors can be different, which will affect the prediction of IDC grade. In terms of data selection, it is necessary to select patient data with an obvious distinction
2. Our model is not combined with other common medical image data such as CT and DR. There are some uncertainties in our model. It needs to combine multiple image data for comprehensive analysis
3. Due to the difficulty of data collection, a large number of labeled data cannot be collected. That is the reason why we did not use the deep neural network model for classification. That is something we need to improve

The above shortcomings will be improved in the next work. Although the experiment still has these shortcomings, it is enough to prove that there is a correlation between the pathological grade of IDC and MRI. Our model can predict the grade of IDC.

## 4. Conclusions

In this paper, we developed a prediction system for the grades of IDC with the highest accuracy of 81.33%. Our model input is the MRI of patients with IDC before operation, and the output of the model is the possible grade of IDC predicted. Our experimental results show that the Gabor wavelet can extract MRI features of IDC patients. There is a certain correlation between the grade of IDC and MRI. The Gabor wavelet analysis combined with the SVM model can solve the problems of small scale medical data and lack of prior knowledge. It has great application value in dealing with the problem of small dataset classification.

## 5. Future Prospects

The next work will be further collecting experimental data and adding experimental samples, not only by collecting data of patients with invasive ductal cancer but also collecting data on their breast tumor types, including breast fibroma and lobular cancer. We hope to get more valuable conclusions by texture analysis combined with pathological results. We also hope to expand the experimental sample by collecting more IDC patient data. By expanding the sample, we try to use the classification method of deep learning [21]. Through the continuous improvement of our model, we can improve the clinical application value of the model.

## Figures and Tables

**Table 1:** Comparative results in generalization accuracy of different kernels and various model parameters (%).

Kernel function	Parameter	Loss function parameter *C*				
		1	2	8	16	32
Linear kernel *k*(*x*, *y*) = *x*^*T*^*y*		77.78	77.77	80.83	71.33	71.67

Polynomial kernel *k*(*x*, *y*) = (*αx*^*T*^*y* + *r*)^*d*^	*d* = 2; *a* = 1/2; *r* = 0	75.83	70.00	75.00	72.50	76.66
	*d* = 3; *a* = 1/2; *r* = 0	69.2	80.83	74.17	71.66	72.50
	*d* = 4; *a* = 1/3; *r* = 0	75.00	75.00	74.16	74.16	75.00
	*d* = 2; *a* = 1/4; *r* = 0	66.66	78.12	75.00	71.66	80.55
	*d* = 3; *a* = 2; *r* = 2	77.77	70.00	78.33	69.16	79.72
	*d* = 4; *a* = 2; *r* = 4	72.91	69.1	71.43	65.83	73.95
	*d* = 6; *a* = 1/6; *r* = 6	72.91	72.91	68.33	76.19	70.83

Gaussian kernel *k*(*x*, *y*) = exp[−‖*x* − *y*‖^2^/2*σ*^2^]	*σ* ^2^ = 0.125	70.83	72.62	80.55	76.66	80.00
	*σ* ^2^ = 0.06	75.00	73.33	80.00	83.33	70.83
	*σ* ^2^ = 5	78.33	76.17	75.00	77.38	70.00
	*σ* ^2^ = 1.6	75.00	76.66	81.33	80.00	77.77

**Table 2:** Results of the *K*-fold cross-validation experiment.

Number of features	Number of folds	Accuracy	Sensitivity	Specificity
3 feature	3	77.78%	76.32%	77.36%
	4	76.39%	73.64%	66.78%
	5	76.00%	68.66%	56.85%

6 features	3	69.4%	85.43%	43.23%
	4	76.37%	83.67%	40.30%
	5	79.85%	85.60%	42.72%

8 features	3	80.55%	90.37%	76.37%
	4	81.94%	86.91%	66.67%
	5	78.47%	89.30%	79.85%

10 features	3	72.22%	77.78%	66.67%
	4	73.61%	75.78%	77.78%
	5	74.21%	69.67%	76.62%

## Data Availability

The clinical data of 30 BC patients MR images were collected from Shandong cancer hospital, choose 28 cases of IDC patients were analyzed. Data are available on request to the authors.

## References

[1] Guo J., Gong G., Zhang B. (2018). miR-539 acts as a tumor suppressor by targeting epidermal growth factor receptor in breast cancer. *Scientific Reports*.

[2] Buys S. S., Sandbach J. F., Gammon A. (2017). A study of over 35,000 women with breast cancer tested with a 25-gene panel of hereditary cancer genes. *Cancer*.

[3] Hwang E. S., Lichtensztajn D. Y., Gomez S. L., Fowble B., Clarke C. A. (2013). Survival after lumpectomy and 254 mastectomy for early stage invasive breast cancer. *Cancer*.

[4] Leithner D., Wengert G. J., Helbich T. H. (2018). Clinical role of breast MRI now and going forward. *Clinical Radiology*.

[5] Lin C. H., Liu C. W., Chen H. Y. (2012). Image retrieval and classification using adaptive local binary patterns based on texture features. *IET Image Processing*.

[6] Zacharaki E. I., Wang S., Chawla S. (2009). Classification of brain tumor type and grade using MRI texture and shape in a machine learning scheme. *Magnetic Resonance in Medicine*.

[7] Nayak D. R., Dash R., Majhi B. (2016). Brain MR image classification using two-dimensional discrete wavelet transform and AdaBoost with random forests. *Neurocomputing*.

[8] Kooi T., Litjens G., van Ginneken B. (2017). Large scale deep learning for computer aided detection of mammographic lesions. *Medical Image Analysis*.

[9] Liu Y. H., Muftah M., Das T., Bai L., Robson K., Auer D. (2012). Classification of MR tumor images based on Gabor wavelet analysis. *Journal of Medical and Biological Engineering*.

[10] Li Z., Mao Y., Li H., Yu G., Wan H., Li B. (2016). Differentiating brain metastases from different pathological types of lung cancers using texture analysis of T1 postcontrast MR. *Magnetic Resonance in Medicine*.

[11] Oulhaj H., Rziza M., Amine A. (2017). Anisotropic discrete dual-tree wavelet transform for improved classification of trabecular bone. *IEEE Transactions on Medical Imaging*.

[12] Huang Z., Lo S., Mayr N., Yuh W. (2013). SU-E-J-108: texture segmentation in magnetic resonance images using discrete wavelet transform combined with Gabor wavelets. *Medical Physics*.

[13] Yuan G., Liu Y., Huang W. (2019). Segmentation of MR breast cancer images based on DWT and K-means algorithm. *Journal of Physics: Conference Series*.

[14] Pisana F., Henzler T., Schönberg S., Klotz E., Schmidt B., Kachelrieß M. (2017). Noise reduction and functional maps image quality improvement in dynamic CT perfusion using a new k-means clustering guided bilateral filter (KMGB). *Medical Physics*.

[15] Guo Y., Li B. Z., Goel N. (2017). Optimised blind image watermarking method based on firefly algorithm in DWT-QR transform domain. *IET Image Processing*.

[16] Javadi S., Hashemy S. M., Mohammadi K., Howard K. W. F., Neshat A. (2017). Classification of aquifer vulnerability using K-means cluster analysis. *Journal of Hydrology*.

[17] Li C., Huang Y., Zhu L. (2017). Color texture image retrieval based on Gaussian copula models of Gabor wavelets. *Pattern Recognition*.

[18] Kay S., Ding Q., Tang B., He H. (2016). Probability density function estimation using the EEF with application to subset/feature selection. *IEEE Transactions on Signal Processing*.

[19] Cinelli M., Sun Y., Best K. (2017). Feature selection using a one dimensional naïve Bayes’ classifier increases the accuracy of support vector machine classification of CDR3 repertoires. *Bioinformatics*.

[20] Yang X., Liu C., Wang Z. (2017). Co-trained convolutional neural networks for automated detection of prostate cancer in multi-parametric MRI. *Medical Image Analysis*.

[21] Renuka S., Annadhason A. (2020). Mil based lung CT-image classification using CNN. *Health and Technology*.

